# Profile of Bioactive Compounds in the Morphological Parts of Wild *Fallopia japonica* (Houtt) and *Fallopia sachalinensis* (F. Schmidt) and Their Antioxidative Activity

**DOI:** 10.3390/molecules24071436

**Published:** 2019-04-11

**Authors:** Sabina Lachowicz, Jan Oszmiański

**Affiliations:** 1 Department of Fermentation and Cereals Technology, Wrocław University of Environmental and Life Science, 37, Chełmońskiego Street, 51-630 Wroclaw, Poland; 2 Department of Fruit, Vegetable and Plant Nutraceutical Technology, Wrocław University of Environmental and Life Science, 37, Chełmońskiego Street, 51-630 Wroclaw, Poland; jan.oszmianski@upwr.edu.pl

**Keywords:** *Fallopia* species, wild plants, polyphenolic compounds, triterpenoids, UPLC-PDA-MS/MS, antioxidative activity

## Abstract

The aim of this study was to determine the content of triterpenoids and polyphenols, and antioxidative activity in leaves, stalks, and roots of plants from the species *Fallopia* as well as to present the main relationship between them. Polyphenolic compounds and triterpenoids were identified with liquid chromatography-photodiode detector-mass spectrometry/quadrupole time of flight (LC-MS-Q/TOF; qualitatively) and quantified with an ultra-performance liquid chromatography-photodiode detector (UPLC-PDA (quantitatively), and their antioxidative activity was determined with radical scavenging capacity (ABTS) and oxygen radical absorbance capacity (ORAC) assays. Generally, the wild *Fallopia japonica* Houtt. species had 1.2 times higher content of bioactive compounds and antioxidative activity than *Fallopia sachalinensis*. Contents of polyphenolic compounds determined in leaves, stalks, and roots were on average 17.81, 10.60, and 9.02 g/100 g of dry weight (DW), whereas the average contents of triterpenoids reached 0.78, 0.70, and 0.50 g/100 g DW, respectively. The leaves were a better source of polymeric procyanidins, phenolic acids, flavones, and flavonols, as well as oleanolic and ursolic acids than the other morphological parts of the tested plants. However, the roots were an excellent source of flavan-3-ols (monomeric and oligomer) and stilbenes, such as resveratrol, and their derivatives. The results obtained showed significant differences between plants of the wild *Fallopia* species and their morphological parts, and enabled selecting the most valuable morphological part of the tested plants to be used for food enrichment and nutraceuticals production. Therefore, the leaves seem to be the best as potential food additives for health, due to the above-average content of polyphenolic compounds and triterpenoids. In turn, roots, with their high contents of stilbenes and polyphenolic compounds, represent a good material for the medical, pharmaceutical, and cosmetic industries. The principal component analysis of the plants of wild *Fallopia* species and their morphological parts confirmed significant differences in their chemical composition.

## 1. Introduction

A growing interest has recently been observed in natural medicine, with phytotherapy being its main branch. According to the World Health Organization (WHO), currently, nearly 80% of the world’s population relies on this form of medicine as part of health care. Herbal medicine is also more widely used by proponents of alternative medicine. According to its practitioners, properly selected and consistently conducted herbal therapy cannot only support the immune system, but even stop the development of a chronic disease. It is for this reason that the focus of this article is on the Japanese knotweed (*Reynoutria japonica,* synonym *Fallopia japonica*) [1].

It should be noted, however, that Japanese knotweed is now considered an invasive species, posing a threat to native wildlife due to its capability to produce substances that inhibit the growth of other plants and make its eradication difficult [2]. Despite its parasitic nature, it is commonly used in phytotherapy. The root of *Fallopia multiflora* (Thunb.), as a wild edible plant, is used to make beverages in Korea [3]. The knotweed herb (*Herba Polygoni cuspidati*) is also used for therapeutic purposes, albeit less frequently, due to the narrower range of its therapeutic effects [4,5]. Roots of *Reynoutria japonica*, which are formally listed in the Chinese Pharmacopoeia [1], are also used in traditional Chinese medicine, owing to their therapeutic effects on diverse inflammatory diseases, tumors, and hepatitis. The most important from the therapeutic point of view of the plants are compounds that belong to the group of polyphenols. Worthy of attention are resveratrol, quercetin, and luteolin [6,7]. Due to the high content of resveratrol in knotweed, its supplementation may have a broad spectrum of activities: neuropoietic (Alzheimer’s disease), antibacterial, antiviral, antimutagenic, antiallergic, as well as inhibiting the accumulation of triglycerides and cholesterol in the liver [4,5,7,8,9]. This plant has also the potential to inhibit *Lyme disease*. According to Goc et al. [8], knotweed supplementation at 200–500 μg/mL reduced the number of *Borrelia burgdorferi* sensu stricto colonies by 30–60%. Research has shown that preparations including knotweed can also be administered to oncological patients. Finally, they are suitable for phytoremediation and phytomelioration of soils contaminated with pesticides, hydrocarbons, and metals [5,6,9].

According to Bralley et al. [4], the coupled action of all knotweed compounds has an interesting effect in relation to the immune system. On one hand, knotweed preparations exhibit immunostimulatory activity, while on the other hand, they inhibit the processes of immunological self-recognition. The better understanding of the potential of the whole plant and its individual parts and their applicability is feasible via thorough analysis of compounds with health-promoting effects. Therefore, the main goal of this study was to evaluate the content of triterpenoids and polyphenols as well as their antioxidative activity in leaves, stalks, and roots of the wild *Fallopia japonica* Houtt. and *Fallopia sachalinensis* (F. Schmidt) species. An additional aim was to determine the main dependencies between the tested material and its phytochemical components using principal components analysis.

## 2. Results and Discussion

### 2.1. Content of Phenolic Compounds

Results of the identification of polyphenolic compounds in plants of wild *F. japonica* (Houtt.) and *F. sachalinensis* (F. Schmidt) (temperate warm climate) and their morphological parts (leaves, stalks, and roots) are shown in Table 1 and Table 2. There were 49 identified polyphenolic compounds belonging to four subclasses—flavan-3-ols, flavones, and flavonols, phenolic acids, and stilbenes. In the leaves, there were 41, in the stalks 42, and in the roots 41 compounds. They were identified by comparison with their authentic standards, mass spectra (MS/MS), retention times (Rt), and their UV spectra reported in the literature (Table 1) [10,11,12,13,14,15]. The analyzed *Fallopia* species had a similar profile of polyphenols, but the content of these compounds differed between species (Table 2).

Polyphenolic compounds are an important group of bioactive compounds found in plants and exhibit various biological activities, including antioxidative, anticarcinogenic, antihypertensive, anti-inflammatory, antiallergic, and antifungal activities [16,17,18]. In our study, the average content of polyphenols in *F. japonica* (Houtt.) reached 13.61 g/100 g DW and was 1.2 times higher than in *F. sachalinensis* (F. Schmidt) (*p* < 0.05) (Table 2). The average content of polyphenols in plants of *Fallopia* species was higher than that in other medicinal plants, such as *Salvia officinalis* (around 2.0 times), *Rosmarinus officinalis* (around 12.0 times), *Tanacetum vulgare* (around 5 times), *Archangelica officinalis* (around 20 times) (grown in temperate climate) (*p* < 0.05) [19], *Melissae folium* (around 6 times), *Spiraea herba* (around 11 times), *Uvea ursi folium* (around 12 times), and *Rubi fructose folium* (around 13 times) (grown in temperate climate) [20]. Contents of phenolic compounds in *Fallopia* species were similar to those determined in Iranian medicinal plants (continental climate), such as *Mellilotus officinalis* [21], and Bulgarian medicinal plants (temperate climate—warm and dry), such as *Cyclopia intermedia*, *Tilia platyphyllos*, Taraxacum officinale complex, and Lavandula angustifolia [22]. Iranian medicinal plants (continental climate), such as *Equisetum maximum*, *Urtica dioica*, and *Adiantum capillus-veneris* [21], and Chinese medicinal plants (continental climate—dryness and tropical monsoon), such as *Rhus chinensis* Mill., *Terminalia chebula* Retz., *Acacia catechu*(L.) Willd., and *Punica granatum* L. [23], have around 2.0, 5.0, 6.0, 2.0, 9.0, 3.0, and 7.0 times lower contents of polyphenols than plants of *Fallopia* species, respectively. The content of polyphenols in plants of wild *Fallopia* species was also higher than in the Algerian medicinal plants (Mediterranean and tropical climate) *Anthemis arvensis* (around 3.4 times), *Artemisia campestris* (around 6 times), and Globularia alypum (around 7 times) [24]. The aforementioned results indicate that knotweed plants are a valuable source of polyphenolic compounds in comparison to other medicinal plants and exhibit various biological properties. Additionally, plants rich in polyphenolic compounds are good materials for the pharmaceutical industry as nutraceuticals or ingredients of dietary supplements and for the food industry as ingredients of functional foods [19]. The highest average content of phenolic compounds was found in leaves of the wild species growing in the natural environmental and reached 17.81 g/100 g DW, which was around 1.7 and 2.0 times higher than in stalks and roots of *Fallopia* species, while in leaves of cultivable *Fallopia* species, the content of polyphenols was 1.6 times lower [15]. According to Lachowicz et al. [17], the level of polyphenols in leaves of wild *Fallopia* species was 8 times higher compared to leaves of *Allium ursinum*. Compared to green and black tea leaves, the content of the tested compounds was about 14 and 12 times higher in leaves of *Fallopia* species. The content of these compounds in leaves and stalks of *F. japonica* (Houtt.) was around 1.4 and 1.2 times higher than in the respective morphological parts of *F. sachalinensis* (F. Schmidt), whereas their content in roots of *F. sachalinensis* (F. Schmidt) was around 1.2 times higher than in roots of *F. japonica* (Houtt.). Roots were also a good source of polyphenolic compounds, whose content was comparable to that found in the roots of *Polygonum Thunb multiflorum* [25]. The differences in the content of polyphenols between the analyzed species depend on many factors, including cultivar, species, morphological parts, and climate [16].

The major group identified in plants of the wild *Fallopia* species and their morphological parts was flavan-3-ols (monomeric, oligomeric and polymeric procyanidins), which on average accounted for 88% of total phenolic compounds. The content of polymeric procyanidins was confirmed using the phloroglucinol method (UPLC-FL). This method proves better in providing more information on the polymeric procyanidins in the plants, because UPLC-PDA-based analyses allow partial detection of the proanthocyanidin fraction. In addition, the profile of flavan-3-ols (monomers and oligomers) was similar to that reported by the other authors [15]. Polymeric procyanidins predominated in leaves and stalks and accounted for 72% and 85%, whereas monomers and oligomers accounted for only 7%. However, higher contents of monomers and oligomers—around 32%—and of polymeric procyanidins—around 60%—were observed in roots. The average content of flavan-3-ols in *F. japonica* (Houtt.) was 11.64 g/100 g DW and was 1.2 times statistically (*p* < 0.05) higher than in *F. sachalinensis* (F. Schmidt) (Table 2). Around 2.3 and 3.8 times higher content of monomers and oligomers was noted in roots than in leaves and stalks. Additionally, around 1.4 and 1.7 times higher content of polymeric procyanidins was determined in leaves than in stalks and roots. The flavan-3-ols, mainly polymeric procyanidins, are very important plant constituents because they affect the flavor of the finished products and exhibit biological activities [26].

The next group identified in plants of wild *Fallopia* species and their morphological parts was flavones and flavonols, represented by fifteen identified compounds. Of these, five flavonols were identified for the first time in the species *Fallopia* compared to the studies of other authors [10,11,12,13,14,15]. Three types of flavone and flavonol derivatives with an MS/MS fragment at *m/z* 301, 285, typical of quercetin, luteolin, and kaempferol derivatives, were found in *Fallopia* plants and their parts. Luteolin derivatives were identified only in leaves of *Fallopia.* Luteolin was identified as -3-*O*-rhamnoside (*m*/*z* 447 due to the loss of 162 Da and *m*/*z* 431 due to the loss of 146 Da) [12,13] (Table 1). Two quercetin derivatives exhibiting a fragment ion at *m*/*z* 301 characteristic for this compound (quercetin) were identified as -3-*O*-rhamno-glucoside (*m*/*z* 609), but only in leaves and stalks of *Fallopia*. Two kaempferol derivatives compared with UV-VIS absorption and the fragment ion at *m*/*z* 285 characteristic for kaempferol were identified as -3-*O*-galactoside, and -3*-O*-glucoside (*m*/*z* 447 due to the loss of 162 Da and *m*/*z* 431 due to the loss of 146 Da) [15] (Table 1). These compounds were mainly located in leaves and accounted for 12% of total phenolics, but in stalks they represented 5% and in roots 0.2% of total phenolic compounds. A higher content of flavones and flavonols was determined in *F. japonica* (Houtt.); it was on average 0.99 g/100 g DW and was around 1.5 times significantly (*p* < 0.05) higher than in *F. sachalinensis* (F. Schmidt) (Table 2). The average content of flavones and flavonols of *Fallopia* species was similar to *Tanacetum vulgare* and higher than in other medicinal plants grown in Poland such as *Salvia officinalis* (around 4.0 times), *Rosmarinus officinalis* (around 1.4 times), and *Archangelica officinalis* (around 1.2 times) [24]. The content of flavones and flavonols in plants of *Fallopia* species was also higher than in Algerian medicinal plants such as *Anthemis arvensis* (around 1.1 times), *Artemisia campestris* (around 1.3 times), and Globularia alypum (around 2.1 times) [24]. The highest content of flavones and flavonols was identified in leaves and it was around 3.6 and 16.0 times higher than in stalks and roots, respectively. Similar results were obtained by analyzing their contents in the morphological parts of *Allium ursinum* [17]. Their content in leaves, stalks, and roots of *F. japonica* (Houtt.) was around 1.6, 1.2, and 17.7 times higher than in the same morphological parts of *F. sachalinensis* (F. Schmidt), respectively. In the analyzed plants, quercetins were the major subclass of flavones and flavonols and represented 97% of their total content, including quercetin 3-*O*-rhamnoside, and quercetin 3-*O*-pentoside that accounted for 73% and 11%, respectively. Quercetin derivatives are important for bodily health, because they have a strong antioxidative activity [16].

The second group identified in plants of *Fallopia* species and their morphological parts were phenolic acids which in their leaves, stalks, and roots accounted for 9%, 2%, and 0.5% of total polyphenolic compounds, respectively. In addition, the profile of phenolic acids was similar to that reported by the other authors [15]. A higher content of phenolic acids was found in *F. japonica* (Houtt.); it reached around 0.66 g/100 g DW and was 1.1 times significantly (*p* < 0.05) higher than in *F. sachalinensis* (F. Schmidt) (Table 2). The average content of flavones and flavonols of *Fallopia* species was higher than in other medicinal plants grown in Poland such as *Salvia officinalis* (around 1.7 times), *Rosmarinus officinalis* (around 1.4 times), and *Archangelica officinalis* (around 1.2 times) [19]. The highest content of phenolic acids was determined in leaves and was around 7.0 and 41.0 times higher than in stalks and roots of the analyzed plants. Similar results were obtained by analyzing the content of these compounds in the morphological parts of *Allium ursinum* [17]. In plants of the analyzed *Fallopia* species, the cis-3-*O*-caffeoylquinic and caftaric acids were major compounds in leaves, and accounted for 29% and 26% of total phenolic compounds, whereas cis-3-*O*-caffeoylquinic and 5-*O*-caffeoylquinic acids were predominant compounds in the stalk (accounting for 32% and 28%), while caftaric and 4,5-Di-*O*-caffeoylquinic acids were the major compounds found in roots (accounting for 21% and 42%). These results corroborated findings reported by Park et al. [27], who stated that an important aspect of the plants was the presence of caffeoylquinic acid, which was the prevailing compound known to affect their flavor.

The last group identified in plants of *Fallopia* species and their morphological parts was represented by stilbenes, which, in their leaves, stalks, and roots, accounted for 0.5%, 1%, and 7% of total polyphenolic compounds, respectively. Among the eight compounds identified, one stilbene was identified for the first time in the species *Fallopia* compared to the studies of other authors [10,11,12,13,14,15]. The compound with Rt = 5.90 and λ = 303 nm having a molecular ion at *m*/*z* 389 and an MS/MS fragment ion at *m*/*z* 227 [M-162-H]^−^ was identified as resveratroloside (Table 1). These compounds were identified based on their standards and available data [10,12,14], and were found only in roots. The reported research indicates that *F. japonica* (Houtt.) and *F. sachalinensis* (F. Schmidt), especially their roots, are an excellent source of stilbenes, mainly resveratrol, which are rarely found in other plants. A higher content of stilbenes was found in *F. japonica* (Houtt.) and was around 1.7 times significantly (*p* < 0.05) higher than in *F. sachalinensis* (F. Schmidt). The average content of piceid and resveratrol in plants of *Fallopia* species was around 1.8 and 1.4 times higher than in Japanese knotweed (the medicinal plant) [12]. Additionally, the content of piceid in grape cv. Casteao from Portugal was around 15 times lower than that in plants of *Fallopia* species and six times lower than in leaves of this plant [28]. The highest content of stilbenes was determined in roots of *Fallopia* species plants and was around 7.4 and 11.1 times higher than in their leaves and stalks. In the analyzed *Fallopia* species, the trans-piceid compounds were major compounds and accounted for 55% of total phenolic compounds. The content of resveratrol and its derivatives, besides flavan-3-ols in roots, can be used as an important indicator of the medicinal potential of plants with respect to their bioactive compounds, nutraceutical value, and also potential use. Additionally, resveratrol is the most important compound of the stilbenes, which are representatives of polyphenolic phytoalexins. They are produced by plants as protective substances against abiotic or biotic stress. Resveratrol and its derivatives offer some health benefits, such as anticarcinogenic, antioxidative, antimicrobial, anti-inflammatory, and antiaging properties [29].

### 2.2. Determination of Tritepenoids

**Figure 1:**
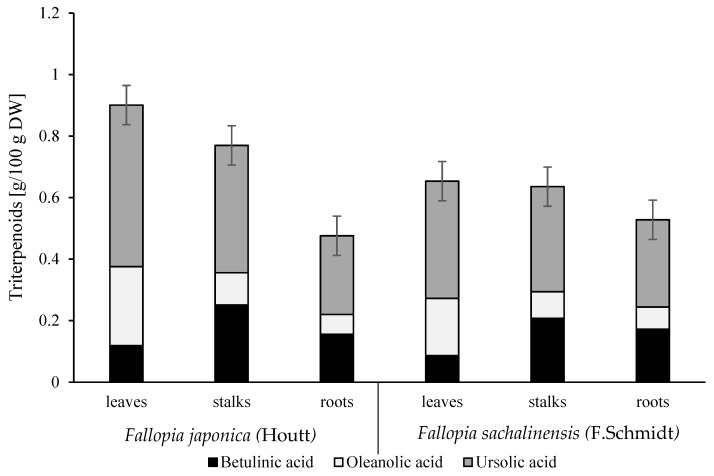
Content of triterpenoids (g/100 g DW) in wild *Fallopia japonica* (Houtt) and *Fallopia sachalinensis* (F. Schmidt) leaves, stalks, and roots.

The outcomes of triterpenoids determination in plants of *F. japonica* (Houtt.) and *F. sachalinensis* (F. Schmidt) and their morphological parts are shown in Figure 1. The analyzed plants of *Fallopia* species, especially their leaves and rhizomes, had a similar profile of triterpenoids to that reported in earlier works [15], whereas contents of these compounds differed between wild and cultivation species [15]. Besides polyphenols, triterpenoids are an important group of bioactive compounds exhibiting biological activities including anticarcinogenic, anti-inflammatory, antifungal and antioxidative ones [16,30,31]. These compounds were identified in stalks and roots of the test plants for the first time ever. The average content of triterpenoids was 0.72 g/100 g DW in *F. japonica* (Houtt.) and was around 1.3 times significantly (*p* < 0.05) higher than in *F. sachalinensis* (F. Schmidt). The highest content of triterpenoids was found in leaves and reached 0.78 g/100 g DW, which was around 1.1 and 1.5 times higher than in stalks and roots, respectively. According to Lachowicz et al. [17], the content of triterpenoids in leaves, stalks, and roots of *Allium ursinum* was around 4, 3, and 1.4 times higher than in the morphological parts of plants of wild *Fallopia* species. In turn, Lachowicz et al. [17] showed a similar content of triterpenoids in the analyzed leaves and their 1.3 times lower content in rhizomes compared to the values determined in leaves in our results. The differences in the content of triterpenoids in the analyzed morphological parts are due to the fact that these compounds are mainly accumulated in the waxy layer of the plants [21,30]. Moreover, their content in plants depends on many factors, including cultivar, degree of maturity, morphological parts, climate, and environmental conditions [31]. The major compound in the analyzed *Fallopia* species was found to be ursolic acid (constituting from 54% to 58% of total triterpenoids), followed by oleanolic acid (from 14% to 29%) and betulinic acid (from 13% to 33%). The ursolic and oleanolic acids prevailed in the leaves; in leaves of *F. japonica* their contents were around 1.4 times significantly (*p* < 0.05) higher than in these of *F. sachalinensis.* Betulinic acid predominated in the stalks. Similar results were obtained by Lachowicz et al. [17] (in *A. ursinum*), Szakiel et al. [30] (in *Prunus avium, Malus domestica)*, and Loza-Mejía and Salazar [16] (in *Olea europaea* leaves).

### 2.3. Antioxidant Activity

**Figure 2:**
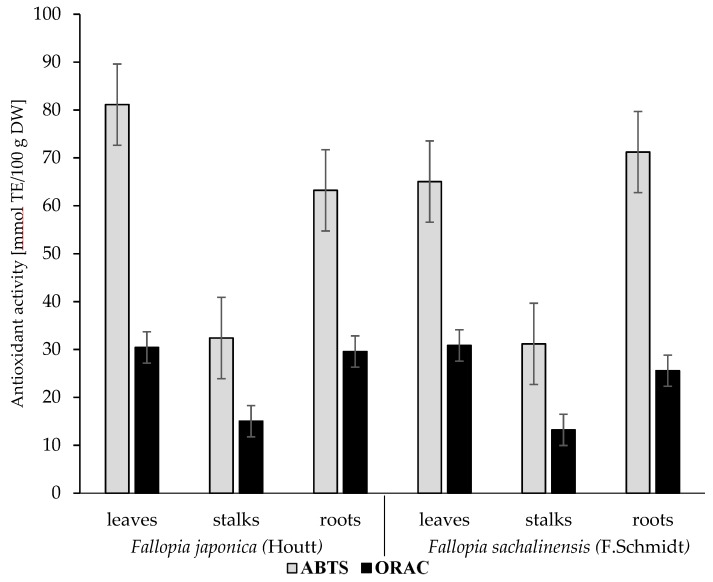
Antioxidative activity determined with in vitro assays: ABTS and ORAC, in wild *Fallopia japonica* (Houtt) and *Fallopia sachalinensis* (F. Schmidt) leaves, stalks, and roots (mmol TE/100 g DW).

The antioxidative activity of foodstuffs is influenced by various mechanisms, and can be determined using different tests pertaining to various mechanisms. Therefore, in vitro assays: radical scavenging capacity (ABTS) and oxygen radical absorbance capacity (ORAC), were used to evaluate the antioxidative activity of *F. japonica* (Houtt.) and *F. sachalinensis* (F. Schmidt) plants and their morphological parts—leaves, stalks, and roots (Figure 2). A significant variation (*p* < 0.05) was found in the antioxidative activity of the analyzed materials. Its average values in *F. japonica* (Houtt.) were 58.91 (ABTS assay) and 24.11 (ORAC assay) mmol Trolox/100 g DW and were around 1.1 times higher than in *F. sachalinensis* (F. Schmidt). The highest antioxidative activity was analyzed in the leaves of *F. japonica* and in the roots of *F. sachalinensis,* i.e., 81.12 and 71.22 (ABTS assay) and 30.42 and 30.85 (ORAC assay) mmol Trolox/100 g DW, respectively. In contrast, the lowest antioxidative activity was measured in the stalks of both *Fallopia* species and reached 31.79 and 14.11 mmol Trolox/100 g DW (ABTS and ORAC assay, respectively) on average. These differences in the antioxidative activity of plants of the analyzed *Fallopia* species and their morphological parts could be attributed to various contents of polyphenolic compounds (Table 2) and triterpenoids (Figure 1). It is generally acknowledged that bioactive compounds, including polyphenolic compounds and triterpenoids, may affect medicinal plants’ antioxidant activity. A strong and positive correlation was found in the analyzed material between the antioxidative activity and contents of total phenolic compounds, phenolic acids, polymeric procyanidins, as well as oleanolic and ursolic acids (*p* < 0.05). In turn, negative correlations were found between contents of betulinic acid and antioxidants (*p* < 0.05). Furthermore, *Fallopia* species presented levels of antioxidants comparable with those of other plants, such as *Rosmarinus officinalis* [19], having high contents of bioactive compounds with proven health benefits. The *Fallopia* species plants presented a higher antioxidative activity than other medicinal plants such as *Melissae folium* (around 2.0 times), *Spiraea herba* (around 3.4 times), *Uvea ursi folium* (around 3.9 times), *Rubi fructose folium* (around 4.3 times) [21], *Salvia officinalis* (around 3.0 times), and *Archangelica officinalis* (around 3.7 times) [19]. Moreover, their leaves and roots exhibited a higher antioxidative activity than other medicinal plants such as *Melissae folium* (around 1.2 times), *Spiraea herba* (around 2.1 times), *Uvea ursi folium* (around 2.4 times), and *Rubi fructose folium* (around 2.6 times) [20].

### 2.4. Principal Component Analysis (PCA)

**Figure 3:**
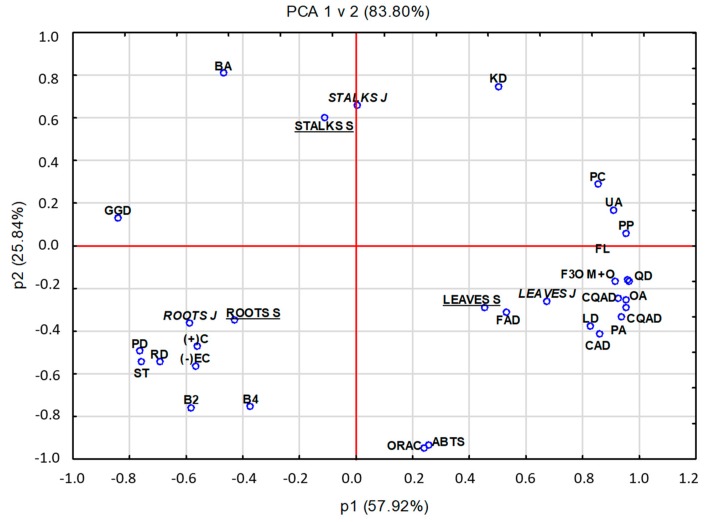
Principal component analysis (PCA) showing the relationship among phenols and antioxidative activity in leaves, stalks, and roots of *Fallopia* species. *Explanation*: BA, betulinic acid; UA, ursolic acid, OA, oleanolic acid; ORAC, oxygen radical absorbance capacity; ABTS, radical scavenging capacity; PP, polymeric procyanidin; PA, phenolic acid; FL, flavonols, PC, phenolic compounds; (+)C, (+)-Catechin; (−)EC, (−)-epicatechin; B2, procyanidin B dimer; B4, procyanidin B tetramer; F3O M+O; flavan-3-ols monomeric and oligomeric; LD, luteolin compounds, RD, resveratrol compounds; ST, total stilbene; PD, piceid compounds; FAD, feruloylquinic acid; CAD, caffeoylquinic acid; CQAD, coumaroylquinic acid; QD, quercetin compounds; KD; kaempferol; GGD, Galloyl glucose compounds; leaves S, roots S, stalk S; parts belonging to *Fallopia sachalinensis* (F. Schmidt); leaves J, roots J, stalk J, parts belonging to *Fallopia japonica* (Houtt).

The PCA showed differences between *Fallopia* species and between leaves, stalks, and roots in their triterpenoids content, polyphenols profiles, and antioxidant activity. Two primary PCs for the study of *Fallopia* species and their parts amounted to 93.80%: i.e., 64.94% for PC1, and 28.88% for PC2 (Figure 3). PC1 illustrated the differences between the content of triterpenoids, polyphenols, and antioxidant activity (ABTS, ORAC), whereas PC2 illustrated the comparison of procyanidins, stilbenes with betulinic acid. The PCA indicated also some differences between the leaves, stalks, and roots of *F. japonica* and *F. sachalinensis*. For example, leaves of both wild *Fallopia* species had the highest antioxidative activity (ABTS and ORAC). Also, leaves had a higher content of oleanolic acid, flavonols, quercetin, lutein derivatives, flavan-3-ols (monomers and oligomers), phenolic acids, feruloylquini, caffeoylquinic, coumaroylquinic, and caftaric acids. Stalks of wild *Fallopia japonica* were characterized by a high amount of kaempferol derivatives, triterpenoids, ursolic acid, and polymeric procyanidins. Stalks of wild *Fallopia sachalinensis* were characterized by a high content of galloyl glucose and betulinic acid. The roots of both wild *Fallopia* species were a good source of stilbenes, piceid and resveratrol derivatives as well as of a procyanidin dimer B, (−)-epicatechin, and a procyanidin tetramer B, (+)-catechin.

Principal component analysis (PCA) has been used earlier to depict correlations between the analytical compounds and cultivars tested. Lachowicz et al. [15] employed PCA to evaluate a correlation between the phenolic, tetraterpenoid, and triterpenoid fractions and the analyzed plants from cultivation *Fallopia* species and their morphological parts. Lachowicz et al. [18] presented the PCA to distinguish bulbs, leaves, flowers, and stems of *A. ursinum* L., based on the phytochemical composition and antioxidative properties. Finally, Sproull et al. [31] used PCA to determine long-term changes in the composition of four herbaceous plants.

## 3. Materials and Methods

### 3.1. Reagent and Standards

Acetonitrile, formic acid, methanol, betulinic, oleanolic and ursolic acid, ABTS (2,2′-azinobis(3-ethylbenzothiazoline-6-sulfonic acid), 6-hydroxy-2,5,7,8-tetramethylchroman -2-carboxylic acid (Trolox), acetic acid, and phloroglucinol were purchased from Sigma-Aldrich (Steinheim, Germany). (−)-Epicatechin, (+)-catechin, chlorogenic acid, neochlorogenic acid, cryptochlorogenic acid, dicaffeic acid, procyanidin B2, *p*-coumaric acid, quercetin-3-*O*-galactoside, quercetin-3-*O*-rhamnoside, caffeic acid, caftaric acid, galloyl acid, and luteolin-3-*O*-galactoside were purchased from Extrasynthese (Lyon, France).

### 3.2. Plant Materials

Leaves, stalks, and roots of wild *Fallopia japonica* (Houtt) and *Fallopia sachalinensis* (F. Schmidt) species were used in the study. The material was divided into morphological parts to check the distribution of the phytochemicals tested. Samples of the growing wild material (~1.0 kg each) were collected at the beginning of September 2018 near the Odra River in Wrocław, Poland (N°51.125988 E°7/08111) (riverside area, highly hydrated).

### 3.3. Determination of Polyphenols

All analyses of polyphenols in the tested samples were carried out using an ACQUITY Ultra Performance LC system (UPLC) equipped with a binary solvent manager (Waters Corp., Milford, MA, USA), a UPLC BEH C18 column (1.7 μm, 2.1 mm × 50 mm, Waters Corp., Milford, MA, USA), and a Q-Tof Micro mass spectrometer (Waters, Manchester, UK) with an ESI source operating in negative and positive modes. The analysis was carried out using full-scan, data-dependent MS scanning from *m*/*z* 100 to 1500. Leucine enkephalin was used as the reference compound at a concentration of 500 pg/μL, and a flow rate of 2 μL/min, and the [M − H]^−^ ion at 554.2615 Da was detected. The [M − H]^−^ ion was detected during 15-min analysis performed within ESI−MS accurate mass experiments, which were permanently introduced via the LockSpray channel using a Hamilton pump. The lock mass correction was ±1.000 for the mass window. The mass spectrometer was operated in the negative-ion mode, set to the base peak intensity (BPI) chromatograms, and scaled to 12,400 counts per second (cps) (100%). The optimized MS conditions were as follows: capillary voltage of 2500 V, cone voltage of 30 V, source temperature of 100 °C, dissolution temperature of 300 °C, and dissolution gas (nitrogen) flow rate of 300 L/h. Collision-induced fragmentation experiments were performed using argon as the collision gas, with voltage ramping cycles from 0.3 to 2 V. The data obtained from UPLC−MS were subsequently entered into the MassLynx 4.0 ChromaLynx Application Manager software.

A protocol described earlier by Lachowicz et al. [15] was followed during the extraction and determination of phenolic compounds. The mobile phase consisted of solvent A (4.5% formic acid, *v*/*v*) and solvent B (100% acetonitrile). The runs were monitored at the following wavelengths: phenolic acids at 320 nm, flavonols at 340 nm, anthocyanins at 520 nm, flavan-3-ols at 280 nm. The PDA spectra were measured over the wavelength range of 200–600 nm in steps of 2 nm. The results were expressed as g/100 g DW.

### 3.4. Determination of Proanthocyanidins

Phloroglucinolysis of samples was performed according to the protocol described by Lachowicz et al. [15]. Phloroglucinolysis products were separated on a Cadenza CD C18 (75 mm × 4.6 mm, 3 μm) column (Imtakt, Japan). Analysis was carried out using a Waters (Milford, MA) system equipped with Waters 474 diode array and scanning fluorescence detectors and Waters 717 plus autosampler. The mobile solvents were 0.25% aqueous acetic acid (A) and acetonitrile (B). Fluorescence detection was monitored at 278 nm and 360 nm. The calibration curves were plotted using (+)-catechin and (−)-epicatechin-phloroglucinol adduct standards. All data were obtained in triplicate. The results were expressed as g/100 g DW

### 3.5. Determination of Triterpenoids

Sample extraction was performed as described by Farneti et al. [32]. Ursolic, oleanolic, and betulinic acids were identified and quantified using the ACQUITY Ultra Performance LC system with a binary solvent manager (Waters Corp., Milford, MA, USA), a UPLC BEH C18 column (1.7 μm, 2.1 mm × 150 mm, Waters Corp., Milford, MA, USA), and a Q-TOF mass spectrometer (Waters, Manchester, UK) equipped with an electrospray ionization (ESI) source, operating in the negative mode. The elution solvent was methanol-acetonitrile (15:85, *v*/*v*), at a flow rate of 0.1 mL min^−1^. The *m*/*z* for betulinic acid was 455.3452, for oleanolic acid 455.3496, and for ursolic acid 455.3365, and the retention times were 6.80, 7.50, and 8.85 min, respectively. The compounds were monitored at 210 nm. All data were obtained in triplicate. The results were expressed as mg/100 g DW.

### 3.6. Determination of Antioxidative Activity

The samples were prepared for analysis as previously described by Lachowicz et al. [15]. The radical scavenging capacity (ABTS) and oxygen radical absorbance capacity (ORAC) methods were used as described by Re et al. [33] and Kapusta et al. [34], respectively. The antioxidative activity was expressed as mmol of Trolox/100 g of DW.

### 3.7. Statistical Analysis

Statistical analysis, one-way ANOVA, and principal component analysis (PCA) were conducted using Statistica version 12.5 (StatSoft, Kraków, Poland). Significant differences (*p* ≤ 0.05) between mean values were evaluated by one-way ANOVA and Duncan’s multiple range test.

## 4. Conclusions

In conclusion, the study results provided complete and important information about the bioactive compounds of plants of wild *Fallopia japonica* Houtt. and *Fallopia sachalinensis* species that were associated with their antioxidative properties. In addition, the analyzed wild *Fallopia* species had a similar profile of polyphenols and triterpenoids, but contents of these compounds in leaves, stalks, and roots were different. Generally, the wild *Fallopia japonica* Houtt. species had a 1.2 times statistically significant higher content of bioactive compounds and antioxidative activity than *Fallopia sachalinensis*. The leaves were a better source of polymeric procyanidins, phenolic acids, flavones, and flavonols, as well as oleanolic and ursolic acids than the other morphological parts of the tested plants. However, the roots were an excellent source of flavan-3-ols (monomeric and oligomer) and stilbenes, such as resveratrol, and their derivatives. Additionally, plants of wild *Fallopia* species and their individual parts may be deemed an attractive plant material and, a good source of many substances with a high health-promoting potential. However, further in vivo and in vitro investigations are necessary to confirm interactions between bioactive compounds. The results obtained showed significant differences between wild *Fallopia* species and their morphological parts, and enabled selecting the most valuable morphological part of the tested plants to be used for food enrichment and nutraceuticals production. Therefore, the leaves seem to the best from the point of view of the food additives to be used as super food and functional food beneficial for health, due to the above-average content of polyphenolic compounds and triterpenoids. In turn, roots, with their high contents of stilbenes and polyphenolic compounds, represent a good material for the medical, pharmaceutical, and cosmetic industries. The principal component analysis of the plants of wild *Fallopia* species and their morphological parts confirmed significant differences in their chemical composition.

## Figures and Tables

**Table 1:** Content of polyphenolic derivatives in wild *Fallopia japonica* (Houtt) and *Fallopia sachalinensis* (F. Schmidt) leaves, stalks, and roots.

Tentative Identification	Retention Time [min]	λ (nm)	Molecular ion MS [H − M]^−^	Fragments MS/MS (*m*/*z*)
Galloyl glucose	1.40	277	331	169
Galloyl glucose	1.45	277	331	169
Galloyl glucose	1.57	277	331	169
3-*O*-caffeoylquinic acid	3.25	324	353	191/179
Cis 3-*O*-caffeoylquinic acid	3.47	326	353	191
Caftaric acid	3.58	328	311	179
Procyanidin dimer B	4.06	279	577	289
Caffeoyl-glucose	4.16		341	179
*p*-Coumaroylquinic acid	4.26	310	337	191/163
(+) Catechin	4.50	279	289	
5-*O*-caffeoylquinic acid	4.62	324	353	191
Cis 5*-O*-caffeoylquinic acid	4.74	324	353	191
Feruloylquinic acid	5.09	320	367	191
Procyanidin dimer B	5.19	279	577	289
Procyanidin dimer B	5.56	279	577	289
*p*-Coumaroylquinic acid	5.68	310	337	191/163
Resveratroloside	5.90	303	389	227
(−)-Epicatechin	6.01	278	289	
Astringin	6.03	328	405	243
Piceatannol 3′-*O*-glucoside	6.14	305	405	243
(+)-Catechin glucoside	6.30	277	451	289
Trans-Piceid	6.35	317	389	227
Procyanidin B gallate	6.61	279	729	577/289
Procyanidin tetramer B	6.72	277	1153	863/575/289
Procyanidin tetramer B	6.88	279	1153	863/575/289
Luteolin 7-*O*-galactoside	7.00	349	447	285
Luteolin 7-*O*-glucoside	7.15	347	447	285
Resveratrol-galleoyl-glucoside	7.22	333	541	227
(+)Catechin gallate	7.31	277	441	289
Quercetin-3*-O*-rhamno-glucoside	7.32	352	609	431/301
Quercetin 3-*O*-rutinoside	7.40	352	609	431/301
Procyanidin tetramer B	7.45	277	1153	863/575/289
Quercetin 3-*O*-galatcoside	7.56	352	463	301
Quercetin 3*-O*-glucoside	7.67	353	463	301
Quercetin 3*-O*-pentoside	7.99	355	433	301
*cis*-Piceid	8.10	285	389	227
Quercetin acetylhexoside	8.20	354	505	463/301
Quercetin 3*-O*-pentoside	8.35	351	433	301
Luteolin 7-*O*-rhamnoside	8.40	342	431	285
Quercetin-3-*O*-rhamnoside	8.51	346	447	301
Kaempferol -3-*O*-galactoside	9.18	345	447	285
Kaempferol -3-*O*-glucoside	9.25	345	447	285
3,4-Di-*O*-caffeoylquinic acid	9.26	324	515	353/191
3,5-Di-*O*-caffeoylquinic acid	9.37	326	515	353/191
4,5-Di-*O*-caffeoylquinic acid	9.49	326	515	353/191
*trans*-Resveratrol	9.51	285	227	
Kaempferol 3*-O*-rhamnoside	9.57	340	431	285
Quercetin	10.84	364	301	
Betulinic acid	6.99		455	
Oleanolic acid	7.66		455	
Ursolic acid	8.38		455	

**Table 2:** Content of polyphenolic derivatives in wild *Fallopia japonica* (Houtt) and *Fallopia sachalinensis* (F. Schmidt) leaves, stalks, and roots.

Group	Compounds	*Fallopia japonica* (Houtt)			*Fallopia sachalinensis* (F. Schmidt)		
		leaves	stalks	roots	leaves	stalks	roots
Flavan-3-ols	Procyanidin dimer B ^3^	0.09 ± 0.01c ^1,2^	0.02 ± 0.00f	0.06 ± 0.00e	0.13 ± 0.00b	0.06 ± 0.00d	0.17 ± 0.00a
	(+) Catechin ^3^	0.19 ± 0.01b	0.08 ± 0.01f	0.09 ± 0.00e	0.14 ± 0.01c	0.09 ± 0.00d	0.22 ± 0.00a
	Procyanidin dimer B ^3^	0.17 ± 0.01e	0.08 ± 0.01f	0.28 ± 0.01b	0.23 ± 0.01d	0.24 ± 0.00c	0.37 ± 0.01a
	Procyanidin dimer B ^3^	0.26 ± 0.01b	0.18 ± 0.01d	0.63 ± 0.02a	0.09 ± 0.01d	0.07 ± 0.01f	0.19 ± 0.00c
	(-)Epicatechin ^3^	0.13 ± 0.01d	0.06 ± 0.00f	0.27 ± 0.01b	0.13 ± 0.01c	0.11 ± 0.00e	0.62 ± 0.01a
	(+)Catechin glucoside ^4^	0.13 ± 0.00b	0.11 ± 0.01d	1.48 ± 0.00a	0.062 ± 0.00f	0.06 ± 0.00d	0.09 ± 0.00c
	Procyanidin B gallate ^4^	0.07 ± 0.00d	0.04 ± 0.00e	0.12 ± 0.01a	0.09 ± 0.00c	0.04 ± 0.00f	0.09 ± 0.00b
	Procyanidin tetramer B ^3^	0.04 ± 0.00c	0.01 ± 0.00f	0.02 ± 0.00e	0.04 ± 0.00b	0.03 ± 0.00d	0.07 ± 0.00a
	Procyanidin tetramer B ^3^	0.06 ± 0.01d	0.02 ± 0.00f	0.09 ± 0.01b	0.07 ± 0.00c	0.03 ± 0.00e	0.17 ± 0.01a
	(+)Catechin gallate ^4^	0.09 ± 0.01b	0.05 ± 0.00e	0.26 ± 0.01a	0.06 ± 0.00d	0.02 ± 0.00f	0.09 ± 0.00c
	Procyanidin tetramer B ^3^	0.07 ± 0.00d	0.04 ± 0.00f	0.14 ± 0.00b	0.13 ± 0.00c	0.07 ± 0.00e	0.17 ± 0.01a
	Procyanidin polymers	15143.39 ± 4.54a	10.05 ± 0.12c	4.24 ± 0.27f	10.62 ± 0.19b	7.97 ± 0.39d	6.72 ± 0.02e
Phenolic acids	Galloyl glucose ^3^	0.01 ± 0.00d^1,2^	0.010.00fa	0.01 ± 0.00b	0.01 ± 0.00e	0.01 ± 0.00a	0.01 ± 0.00c
	Galloyl glucose ^3^	0.01 ± 0.00f	0.01 ± 0.00d	0.01 ± 0.00	0.01 ± 0.00b	0.01 ± 0.00c	0.01 ± 0.00e
	Galloyl glucose ^3^	0.01 ± 0.00d	0.01 ± 0.00a	0.01 ± 0.00c	0.01 ± 0.00f	0.01 ± 0.00e	0.01 ± 0.00b
	3-*O*-caffeoylquinic acid ^3^	0.03 ± 0.01b	0.01 ± 0.00c	0.00 ± 0.00d	0.04 ± 0.00a	0.01 ± 0.00c	0.01 ± 0.00c
	Cis 3-*O*-caffeoylquinic acid ^3^	0.49 ± 0.01a	0.07 ± 0.00c	0.01 ± 0.00f	0.45 ± 0.01b	0.07 ± 0.01d	0.01 ± 0.00e
	Caftaric acid ^3^	0.44 ± 0.00b	0.02 ± 0.00d	0.01 ± 0.00e	0.58 ± 0.01a	0.02 ± 0.00c	0.00 ± 0.00f
	Caffeoyl-glucose ^4^	0.02 ± 0.00a	0.01 ± 0.00c	0.01 ± 0.00e	0.04 ± 0.00b	0.01 ± 0.00d	0.01 ± 0.00f
	*p*-Coumaroylquinic acid ^3^	0.23 ± 0.01a	0.04 ± 0.00c	0.01 ± 0.00f	0.13 ± 0.01b	0.02 ± 0.00d	0.01 ± 0.00e
	5-*O*-caffeoylquinic acid ^4^	0.30 ± 0.00a	0.08 ± 0.00c	0.01 ± 0.00e	0.25 ± 0.01b	0.06 ± 0.01d	0.01 ± 0.00f
	Cis 5-*O*-caffeoylquinic acid ^3^	0.022 ± 0.00a	0.01 ± 0.00c	0.01 ± 0.00e	0.02 ± 0.00b	0.01 ± 0.00d	0.01 ± 0.00f
	3-*O*-*p*-Coumaroylquinic acid ^3^	0.01 ± 0.00a	0.00 ± 0.00e	nd	0.01 ± 0.00b	0.01 ± 0.00d	0.01 ± 0.00c
	Feruloylquinic acid ^3^	0.01 ± 0.00c	0.01 ± 0.00e	nd	0.02 ± 0.00a	0.01 ± 0.00d	0.01 ± 0.00b
	3-*O*-*p*-Coumaroylquinic acid ^3^	0.09 ± 0.00a	0.03 ± 0.01c	nd	0.03 ± 0.00b	0.01 ± 0.00d	0.01 ± 0.00e
	3,4-Di-*O*-caffeoylquinic acid ^4^	nd^≠^	nd	nd	nd	nd	0.01 ± 0.00a
	3,5-Di-*O*-caffeoylquinic acid ^4^	nd	nd	nd	nd	nd	0.01 ± 0.00a
	4,5-Di-*O*-caffeoylquinic acid ^4^	nd	nd	nd	nd	nd	0.03 ± 0.00a
Flavones and Flavonols	Luteolin 7-*O*-galactoside ^4^	0.02 ± 0.00b ^1,2^	nd	nd	0.05 ± 0.00a	0.01 ± 0.00c	nd
	Luteolin 7-*O*-glucoside ^3^	0.01 ± 0.00b	nd	nd	0.03 ± 0.00a	0.01 ± 0.00c	nd
	Luteolin 7-*O*-rhamnoside ^3^	0.03 ± 0.00a	nd	nd	0.02 ± 0.00b	nd	nd
	Quercetin-3-*O*-rhamno-glucoside ^4^	0.02 ± 0.00a	0.02 ± 0.00c	nd	0.01 ± 0.00d	0.02 ± 0.00b	nd
	Quercetin 3-*O*-rutinoside ^3^	0.03 ± 0.00a	0.02 ± 0.00d	0.01 ± 0.00e	0.02 ± 0.00c	0.02 ± 0.00b	nd
	Quercetin 3-*O*-galactoside ^3^	0.05 ± 0.00a	0.02 ± 0.00c	0.01 ± 0.00e	0.03 ± 0.00b	0.02 ± 0.00d	0.01 ± 0.00f
	Quercetin glucoside ^3^	0.105 ± 0.01b	0.07 ± 0.00d	nd	0.12 ± 0.01a	0.09 ± 0.00c	nd
	Quercetin pentoside ^4^	0.09 ± 0.00a	0.02 ± 0.00d	0.01 ± 0.00e	0.05 ± 0.00b	0.03 ± 0.00c	nd
	Quercetin acetylhexoside ^4^	0.08 ± 0.00a	0.04 ± 0.00d	0.01 ± 0.00e	0.05 ± 0.00c	0.06 ± 0.00b	nd
	Quercetin pentoside ^4^	0.25 ± 0.08a	0.08 ± 0.00d	0.01 ± 0.00	0.17 ± 0.01b	0.09 ± 0.00c	nd
	Quercetin rhamnoside ^4^	1.71 ± 0.01a	0.28 ± 0.00c	0.01 ± 0.00e	0.89 ± 0.02b	0.16 ± 0.01d	nd
	Kaempferol -3-*O*-galactoside ^3^	0.01 ± 0.00a	0.01 ± 0.00a	0.01 ± 0.00a	0.01 ± 0.00a	0.01 ± 0.00a	nd
	Kaempferol -3-*O*-glucoside ^3^	0.01 ± 0.00a	0.01 ± 0.00a	nd	0.01 ± 0.00a	0.01 ± 0.00a	nd
	Kaempferol -3-*O*-rhamnoside ^4^	nd	nd	nd	0.01 ± 0.00a	0.01 ± 0.00b	nd
	Quercetin ^4^	nd	nd	nd	0.04 ± 0.00a	0.02 ± 0.00b	nd
Stilbene	Resveratroloside ^4^	nd	nd	0.02 ± 0.00b	nd	nd	0.03 ± 0.00a
	Astringin ^3^	nd	nd	0.03 ± 0.00b	nd	nd	0.05 ± 0.00a
	Piceatannol 3′-*O*-glucoside ^4^	0.02 ± 0.00c ^1,2^	0.01 ± 0.00d	0.22 ± 0.01a	0.02 ± 0.00c	0.01 ± 0.00d	0.05 ± 0.00b
	Trans-Piceid ^3^	0.03 ± 0.00e	0.04 ± 0.01d	0.50 ± 0.01a	0.02 ± 0.00f	0.06 ± 0.00c	0.22 ± 0.00b
	Resveratrol-galleoyl-glucoside ^4^	nd	nd	nd	nd	nd	0.02 ± 0.00a
	Cis-Piceid ^3^	0.01 ± 0.00c	0.01 ± 0.00c	0.02 ± 0.00b	0.01 ± 0.00c	0.01 ± 0.00b	0.05 ± 0.00a
	Trans-Resveratrol ^3^	0.01 ± 0.00b	0.01 ± 0.00b	0.02 ± 0.00a	0.01 ± 0.00b	0.01 ± 0.00b	0.02 ± 0.00a

^1^ a–e Means ± SD followed by different letters within the same line represent significant differences (*p* < 0.05). Data are the averages of triplicates; ^2^ Values are means ± standard deviation. *n* = 3; ^3^ Identification confirmed by commercial standards; ^4^ Identification by comparison of MS data with the literature and their identification is tentative.
